# Detecting clinically relevant new information in clinical notes across specialties and settings

**DOI:** 10.1186/s12911-017-0464-y

**Published:** 2017-07-05

**Authors:** Rui Zhang, Serguei V. S. Pakhomov, Elliot G. Arsoniadis, Janet T. Lee, Yan Wang, Genevieve B. Melton

**Affiliations:** 1 Institute for Health Informatics, University of Minnesota, Minneapolis, MN USA; 2 Department of Surgery, University of Minnesota, Minneapolis, MN USA; 3 College of Pharmacy, University of Minnesota, Minneapolis, MN USA

**Keywords:** Natural language processing, Electronic health records, Statistical language models, Semantic similarity, New information, Redundancy, Clinical specialty

## Abstract

**Background:**

Automated methods for identifying clinically relevant new versus redundant information in electronic health record (EHR) clinical notes is useful for clinicians and researchers involved in patient care and clinical research, respectively. We evaluated methods to automatically identify clinically relevant new information in clinical notes, and compared the quantity of redundant information across specialties and clinical settings.

**Methods:**

Statistical language models augmented with semantic similarity measures were evaluated as a means to detect and quantify clinically relevant new and redundant information over longitudinal clinical notes for a given patient. A corpus of 591 progress notes over 40 inpatient admissions was annotated for new information longitudinally by physicians to generate a reference standard. Note redundancy between various specialties was evaluated on 71,021 outpatient notes and 64,695 inpatient notes from 500 solid organ transplant patients (April 2015 through August 2015).

**Results:**

Our best method achieved at best performance of 0.87 recall, 0.62 precision, and 0.72 F-measure. Addition of semantic similarity metrics compared to baseline improved recall but otherwise resulted in similar performance. While outpatient and inpatient notes had relatively similar levels of high redundancy (61% and 68%, respectively), redundancy differed by author specialty with mean redundancy of 75%, 66%, 57%, and 55% observed in pediatric, internal medicine, psychiatry and surgical notes, respectively.

**Conclusions:**

Automated techniques with statistical language models for detecting redundant versus clinically relevant new information in clinical notes do not improve with the addition of semantic similarity measures. While levels of redundancy seem relatively similar in the inpatient and ambulatory settings in the Fairview Health Services, clinical note redundancy appears to vary significantly with different medical specialties.

## Background

One key potential advantage of electronic health record (EHR) adoption is the opportunity for health care organizations to leverage the EHRs for organizational initiatives around improving patient care quality, decreasing costs, and increasing operational efficiency. Despite these possible benefits, EHRs also unfortunately bring some unwanted secondary consequences. Most EHRs allow copy/paste functionality and automated importing of clinical information in other parts of the record to improve the efficiency of creating and editing clinical notes within a time-constrained clinical environment. Bringing in preexisting information from different parts of the EHRs for creating notes results in the repetition of patient information within notes. Overall, new notes often contain a mixture of recombinant versions of previous notes [1] and re-copy of structured information within other parts of the chart (e.g., medication list). Copy/paste also allows for something that might change over time, like the review of systems (containing the patient’s signs and symptoms) or physical examination, to be directly copied, which potentially results in errors or communication of inaccurate information in subsequent notes [2]. Overall, this leads to long, redundant, and potentially inaccurate EHR notes with irrelevant or obsolete information. As a result, the process of reading and reviewing patient notes for applications like direct patient care, quality improvement, or clinical research can be difficult and laborious.

Irrelevant and redundant information both increases the length of notes and decreases a reader’s ability to identify new information. According to a recently published American Health Information Management Association (AHIMA) report, between 74 and 90% of physicians use the copy/paste function, and 20–78% of physician notes are redundant text [3]. The mixture of redundant and new information also increases the associated cognitive load when synthesizing patient notes, especially in cases where patients have complex conditions and medical histories [4]. A follow-up study demonstrated that applying a visualization cue (highlighting with different color text) to new information as a feature of a prototype user interface saved time for providers reviewing notes and improved note navigation [5].

With respect to outdated and incorrect information in clinical notes, propagation of errors in clinical notes ultimately damages the trustworthiness and integrity of clinical notes in the EHR. For example, in one case report, the same reference to the gastroenterologist was incorrectly repeated by many physicians in several departments during the course of 7 years [2]. In another case reported in the Agency for Healthcare Research and Quality (AHRQ) WebM&M, information in the admission note that the patient would receive heparin to prevent venous thromboembolism was copied and pasted for 4 consecutive hospital days, leading to failure of the providers to order the appropriate thromboembolic prophylaxis [6]. This resulted in the patient being re-hospitalized and diagnosed with a pulmonary embolism 2 days later [6]. In addition to anecdotal case reports, others have demonstrated that the existence of redundant information leads to decreased use of clinical notes by clinicians [7]. Moreover, it has been suggested that redundancy in the EHR corpus must be accounted for when conducting text mining tasks [8] and that detecting semantic redundancy remains one of six challenges for EHR summarization [9]. Therefore, appropriate automated solutions are needed for this problem.

Additionally, methods for automated identification of new information may be valuable as a feature in EHR systems and may help support clinical practice. In one study, visualization of new information in clinical notes appeared to positively influence the synthesis of patient information in EHR documentation by clinicians and may save clinicians time when synthesizing patient information [5]. Thus, high quality automated methods for new information detection may be important foundational technology for creating effective visualization of clinical documents within EHR systems.

In this study, we evaluated the incorporation of semantic similarity techniques to our previously described approach based on *n*-gram language models [10] and compared note redundancy (i.e., counterpart of the relevant new information) across various specialties and settings.

### Related work

A number of approaches have previously been reported around quantifying redundancy in clinical notes. For example, Weir et al., manually reviewed 1,891 notes in the Salt Lake City Veterans Affairs (VA) health care system and found that approximately 20% of notes contained copied text [11]. With respect to automated methods, 167,076 progress notes for 1,479 patients from the VA Computerized Patient Record System (CPRS) were examined using pair-wise comparison of all patient documents to identify matches of at least 40 consecutive word sequences in two documents. They found 9% of progress notes contained copied text [12]. Wrenn et al. used global alignment to quantify the percentage of redundant information in a collection of 1,670 inpatient notes (including sign-out note, progress note, admission note and discharge note) and found an average of 78% and 54% redundant information in sign-out and progress notes, respectively [13]. More recently, Cohen et al. used Smith-Waterman text alignment algorithm to quantify redundancy both in terms of word and semantic concept repetition [8]. They found that corpus redundancy had a negative impact on the quality of text-mining and topic modeling, and suggested that redundancy of the corpus must be accounted for in applying subsequent text-mining techniques for many secondary clinical applications [8].

Other work has looked at techniques using a modification of classic global alignment with a sliding window and lexical normalization [14]. This work demonstrated a cyclic pattern in the quantity of redundant information longitudinally in ambulatory clinical notes for a given patient, and that the overall amount of redundant information increases over time. Subsequently, statistical language models were used to identify and visualize relevant new information via highlighting texts [15]. Work quantifying information redundancy between each note also demonstrates that, in most cases, clinicians tend to copy information exclusively from the most recent note. New information proportion (i.e., percentage of counterpart of redundant information) also appears to be a helpful metric for navigating clinicians or researchers to notes or information in notes that is clinically significant [16]. Moreover, categorizing clinically relevant new information based on semantic type group [17] (e.g., medication, problem/disease, laboratory) can potentially improve information navigation for specific event types [10].

### Statistical language models and semantic similarity

Statistical language modeling (SLM) is widely used in NLP and text-mining tasks, such as parsing, part-of-speech tagging, and information retrieval [18, 19]. SLM assigns a probability to a set of *n* words based on a probability distribution from a specific corpus. To simplify the calculation, often the *Markov* assumption is applied so that only a few nearby words affect the next word. Due to the sparseness of the corpus, a probability of zero is assigned to the unseen *n*-gram, making zero propagated to the whole string. To avoid this, a smoothing method (e.g., Laplace smoothing) is typically used to redistribute the probability.

Semantic similarity metrics aim to measure the semantic likeness between two biomedical concepts by determining the closeness of concepts using different measures such as closeness in a hierarchy or information content-based metrics. High quality semantic similarity metrics within the biomedical domain are critical for improving the performance of information retrieval functions and NLP information extraction tasks. In the context of automated measurement of information redundancy, measures of semantic similarity may be useful to perform semantic normalization between pieces of text that are being compared to determine the degree of redundancy. For example, it may be useful to treat orthographically different but semantically synonymous or similar terms as equivalent (e.g., heart vs. cardiac) when comparing two texts to identify new information, thus potentially increasing accuracy of these associated methods.

In the clinical domain, several methods have been developed to measure semantic similarity based on various types of relationships between concepts, such as broader/narrower [20], parent/child [21] and is-a [22, 23] relations. The Unified Medical Language System (UMLS)::Similarity package was developed based on UMLS relationships. It provides an open-source framework for measuring semantic similarity and comparing results using various methods [24]. Semantic similarity measures are generally classified into two categories: path-based measures and information content (IC)-based measure. The IC of a concept is defined as negative the log likelihood of *P*(*c*), *-logP*(*c*), where *P*(*c*) is the probability of the concept *c* [25]:

1. Resnik defined the similarity of two concepts as the IC of their least common subsumer (LCS) [25]:
  $$ scor{e}_{res}\left({c}_1,{c}_2\right) = I C\left( lcs\left({c}_1,{c}_2\right)\right) $$
2. Jiang and Conrath used the IC of each individual concept and their LCS to estimate similarity [26]:
  $$ scor{e}_{jcn}\left({c}_1,{c}_2\right)=\frac{1}{IC\left({c}_1\right)+ IC\left({c}_2\right)-2\times IC\left( lcs\left({c}_1,{c}_2\right)\right)} $$
3. Lin extended the similarity score as [27]:
  $$ scor{e}_{lin}\left({c}_1,{c}_2\right)=\frac{2\times IC\left( lcs\left({c}_1,{c}_2\right)\right)}{IC\left({c}_1\right)+ IC\left({c}_2\right)} $$

Compared to path-based similarity measures, IC-based measures are relatively computationally expensive, but have been demonstrated to provide statistically significantly higher accuracy compared to path-based measures [28]. In this study, we only investigated the performance of semantic similarity by applying these three IC-based measures.

### Clinical specialty or subject matter domain

Subject matter domain (SMD) is roughly equivalent to clinical specialty (e.g., cardiology, psychiatry) and is one of the five axes in the HL7/LOINC document ontology (DO): Kind of Document (KOD), Type of Service (TOS), Setting, SMD and Role [29]. The SMD axis was expanded by Shapiro et al. using the value list of the American Board of Medical Specialties and SMD values from one institution’s notes [30]. Recently, we have evaluated the adequacy of DO to represent document data in our healthcare institution’s Clinical Data Repository [31]. We used these specialty mappings in the current study to explore differences by various SMD providers in the amount of redundant and irrelevant information in clinical notes as well as explore the utility of sematic similarity metrics.

## Methods

**Fig. 1:**
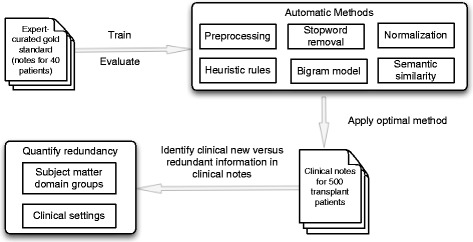
Overview of methods

The methodological approach for this study includes: (1) develop expert-curated gold standard; (2) develop and evaluate automatics methods; (3) apply the best method to identify clinically relevant new information in clinical notes; and (4) quantify and compare redundancy across various SMD groups and clinical settings (Fig. 1).

### Data collection

Longitudinal electronic clinical notes were retrieved from the University of Minnesota affiliated Fairview Health Services hospitals and clinics clinical data repository. To develop the method, we randomly selected 40 patients with multiple co-morbidities, allowing for relatively large numbers of longitudinal records in the outpatient clinic setting. These notes were extracted in text format from the Epic™ EHR system during a six-year period (06/2005–6/2011) and arranged chronologically for each individual patient. We limited our study to progress notes authored by providers including physicians, residents, and advanced practice providers. Institutional review board approval was obtained and informed consent waived for this minimal risk study.

To further compare different levels of redundancy in notes by various SMDs, another set of clinical notes from 500 randomly-selected recent solid organ transplant patients were collected (April through August 2015) to ensure that patients were cared for by a wide range of specialists. Relevant note metadata including note time, encounter ID, and specialty author were also extracted.

### Manually reviewed annotation as gold standard

Four 4^th^ year medical students (two male and two female) were asked to use their clinical judgment to identify clinically relevant new information within each patient document, starting from the second document during an inpatient stay (the first note reviewed was typically the History & Physical Exam note), then moving forward chronologically in each patient’s set of notes new information was identified. The General Architecture for Text Engineering (GATE) software was used to annotate new information in clinical notes. GATE allows for the creation of a customized annotation schema for the annotation of text and XML outputs through a graphical user interface. Each annotator first annotated a single patient record, referring to established annotation guidelines as described previously [32]. Annotators then met to compare one another’s annotations and reach a consensus on the annotation schema. After reaching consensus and updating annotation guidelines, they annotated a common set of 40 patient records. Fleiss’s Kappa statistic and percentage agreement were used to evaluate inter-rater agreement at a sentence or statement level.

To analyze the disagreement between annotators and achieve a high-quality gold standard, a resident physician (EA) manually reviewed all annotations to judge the disagreement between annotators. Overall, we created a reference standard with 591 progress notes over 40 inpatient admissions (median 13 (range 8–28)) provider notes per admission). We then compared the automatically computed redundancy results with revised annotations. Four hundred notes were used to train the models and the remaining notes were used for evaluating the methods. Performance of automated methods was compared to the reference standard and precision, recall, and F-measure was reported at a sentence or statement level.

### Automated method

We developed different *n*-gram models with and without semantic similarity algorithms. We only focused on bigram models since bigram models outperformed other *n*-gram models in our prior study [10]. The methods in this study included six steps: 1) text preprocessing; 2) removal of classic stopwords [33] and term frequency - inverse document frequency (TF-IDF) stopwords; 3) lexical normalization; 4) application of heuristic rules to classify clinical relevance; 5) build a baseline model with smoothing algorithms; 6) modification of baseline with three semantic similarity algorithms when compared with biomedical terms. The details of these steps are as follows:

- All progress notes were chronologically ordered and grouped for each individual inpatient admission. These notes were separated into sentences or sections. MetaMap [34], a tool mapping input texts to UMLS concepts, was used to extract biomedical terms.
- Both classic stopwords and stopwords defined by optimal threshold of TF-IDF distribution based on the entire note corpus were removed. This step deemphasizes these less important words for building the language models.
- To normalize lexically different forms of the same term as equivalent, lexical variant generation (LVG) was used when building bigram models.
- Clinical relevance was judged based on developed heuristic rules previously described using information about factors such as section content, clinical note headers, and signature lines [15]. For example, vital signs in all notes are judged as relevant new information. All note headers and footer are treated as clinically irrelevant information.
- A bigram language model was built with all previous notes for each individual patient. The probability of the bigrams with Laplace discounting algorithms was calculated and an optimal threshold value was used to identify new information in the target progress notes. This was then used as a baseline model for further applications.
- MetaMap was used to map biomedical terms to UMLS concepts. Using the UMLS::Similarity package, three semantic similarity algorithms (i.e., Resnik, Jiang and Conrath, and Lin) were applied. The similarity score between two concepts is in the range of 0 to 1. Thus, we experimented different semantic similarity thresholds (0.5 to 1.0 with an interval of 0.1) to consider as equivalent when comparing two terms. We then selected an optimal threshold for each algorithm with the best F-measure. The optimal threshold value was used to identify how similar two biomedical terms were to one another. We focused only on two concepts in the same context (e.g., “pain is better” and “chest pain is better”).

### SMD categorization

SMDs were classified into categories of Internal Medicine, Surgery, Pediatric, or Psychiatry and into general and subspecialty services. As such, Internal Medicine was further classified into general services (e.g., “General Medicine”, “Internal Medicine”) or subspecialty services (e.g., “Gastroenterology”, “Nephrology”). Diagnostic specialties (e.g., “Pathology”, “Radiology”) and subspecialties (“Blood Banking and Transfusion”) were also classified, although progress notes from these areas appeared infrequently compared to other areas. Non-physician specialties (e.g., “Pharmacy”, “Occupational Therapy”) were categorized as such. This classification was first created by a resident physician (EA) and then evaluated separately by a staff physician (GM). Disagreements were discussed and resolved by consensus.

### Redundancy quantification across SMDs

In total, we collected 71,021 outpatient notes and 64,695 inpatient notes from 2,758 inpatient encounters from randomly selected 500 solid organ transplant patients. We grouped all outpatient notes for individual patients together for the analysis, but kept inpatient notes in the same encounter as separate groups to conduct the redundancy analysis. We applied the Baseline + Lin language model to conduct analysis described in the “Automated Method” and quantified the percentage of redundancy defined as (the number of sentences without new information)/(the number of all sentences) in a single note. We then reported means and standard deviations of redundancy percentages for categorized specialties.

## Results

### Annotation evaluation and model performance

The four raters showed reasonable agreement on the task of identifying new information on the overlapped annotation. Fleiss Kappa coefficient of the four annotators was 0.53 (percentage agreement 0.68) on clinically relevant new information identification at the sentence/statement level, considered moderately good.

We compared results generated by our automated approach with the refined reference standard. The precision, recall, F-measure, and optimal threshold of these methods are summarized in Table 1. We found that the all three models with semantic similarity measures improve the recall compared with the baseline model, although no significant differences in F1-measure were found. In the case of detecting new information in clinical notes, recall is very important, as clinicians would like a tool to retrieve any clinically relevant patient information.

**Table 1:** Performances of various models compared with reference standard

Model	Recall	Precision	F1-Measure	Optimal threshold
Baseline	0.85	0.64	0.73	-
Baseline + Lin	0.87	0.62	0.72	0.9
Baseline + Res	0.87	0.61	0.72	0.9
Baseline + Jcn	0.87	0.61	0.72	0.9

### SMD categorization and percentage of redundancy across SMDs

The quantity of redundancy and irrelevant information in notes by various SMDs, classified according to our previously described categorizations, is listed in Table 2. We found that inpatient notes have higher redundancy (68.3%) compared to outpatient progress notes (60.7%). The redundancy for SMDs ranged from 25.50% (Inpatient Radiology notes) to 76.29% (Outpatient Pediatrics notes). Comparing the level of redundancy according to SMD category, top three specialties include Pediatrics (74.88%), followed by Psychiatry (84.17%) and Specialty Internal Medicine (67.51%). Radiology (45.86%), Emergency Medicine (47.26%), and Surgery (55.05%) have lower redundancy percentages.

**Table 2:** Redundancy percentages in clinical notes by various SMD groupings

SMD Group	Overall Redundancy % (Standard Deviation)	Inpatient Note Redundancy % (Standard Deviation)	Outpatient Note Redundancy % (Standard Deviation)
Overall	64.31% (36.49%)	68.30% (33.80%)	60.68% (38.42%)
Emergency Medicine	47.26% (40.85%)	41.83% (42.80%)	54.06% (37.18%)
Critical Care Medicine	67.82% (34.04%)	67.82% (34.04%)	--
Internal Medicine Overall	65.63% (35.64%)	70.11% (32.30%)	62.50% (37.50%)
Specialty Internal Medicine	67.51% (35.10%)	72.05% (31.23%)	65.00% (36.83%)
Surgery	55.05% (39.87%)	55.04% (39.73%)	55.06% (39.73%)
Pediatrics	74.88% (30.47%)	67.00% (37.11%)	76.29% (28.90%)
Psychiatry	57.12% (39.86%)	56.80% (40.30%)	61.46% (33.50%)
Radiology	45.86% (40.76%)	25.50% (35.62%)	64.86% (35.78%)

## Discussion

This study focuses on the development of methods to address an issue that is widely recognized as a problem in EHR documentation – copying and pasting – and its resultant redundant information in clinical notes that affects the analysis of these notes for downstream applications and use for clinical care. Several attempts have been made to quantify the redundant information in clinical notes [10, 13, 32, 35]. Most of the methods only identify redundant information on a lexical level and do not attempt to detect clinically relevant new information resulting from semantic similar terms and associated automated techniques. Along with exploring differences in redundancy by different specialty authors, one of the objectives in this study was to explore the possibility of using semantic similarity techniques for improving the performance of our approach using statistical language models.

Semantic similarity measures usually are used to detect the relative difference in meaning of different concepts. In this pilot study, we used these metrics to judge if two biomedical concepts have similar meaning in the same context (such as “switch A to B” versus “change A to B”). However, we found limited instances of these variations in clinical notes; clinicians either copy/paste the sentences directly or rephrase the meaning of the sentence from one section (add or discount a drug to the medication list) to another section in different format (e.g., state the added drug), instead of simply changing a few words. As such, none of the three semantic similarity algorithms significantly improve the performance for identifying clinically relevant new information. However, all three methods increase the recall compared with the baseline method. Recall is the more important in this application since we do not want to miss any important information for clinicians for clinical decision-making.

Overall, our methods have relatively high recall values and somewhat lower precision, since the statistical language models are very sensitive to small changes in the sentences. Classifying the clinical relevance of the newly written information in the target note remains a challenging task. Formatting issues, such as header note information, signatures, as well as some negative exam results, can be trained from notes. Sometimes, other infrequent phrases such as “face to face time is 15 min” could bring false positives. Another large source of false positives was from sentences with vague relevance, such as “No acute events overnight”. One could think this is irrelevant since the patient did not experience any new events. However, one can also treat this as important information since the patient’s return to normalcy may be an important turning point in care. Thus, the relevance of a statement also relates to the clinical context.

We also investigated redundancy levels in various clinical specialty groups. To our knowledge, this is the first study to report percentage of redundant information of notes (including both inpatient and outpatient notes) authored by various specialties. Interestingly, most specialties have a relatively high redundancy (over 60%) although some have lower percentages (e.g., Radiology, Surgery). Although in total we included over 135,000 notes in the study, we did not cover all the specialties in HL7/LOINC DO with our analysis of a group of solid organ transplant patients. In a prior study, we found relatively high redundancy levels (76%) in a smaller sample of (100 inpatient and 90 outpatient) notes [35]. In our current study, we found inpatient notes had a higher but relatively similar redundancy percentage (68%) compared to outpatient notes (61%) over a larger corpus. When comparing notes by clinical settings and the specialty of the author, inpatient notes appear have greater (e.g., Internal Medicine), similar (e.g., Surgery) or less (e.g., Pediatrics, Radiology) redundancy than outpatient notes.

The current study is limited by being conducted at a single institution and by the challenges, as noted, with the annotation process for creating a reference standard. Our future plans are to develop an efficient classifier for clinical relevance. It may also be worthwhile to explore differences in opinion with respect to different roles (e.g., physicians, nurses) and specialties on the utility of new information visualization cues for clinical notes.

## Conclusions

We investigated the use of semantic similarity with statistical language models for information navigation with longitudinal clinical notes and compared differences in redundancy by setting and specialty. Semantic similarity measures help to improve recall of language models to detect clinically relevant new information. Quantifying redundancy in over 135,000 clinical notes by automatic methods, we found outpatient and inpatient notes had 60.7% and 68.3% redundancy, respectively. When comparing differences by specialty author, note redundancy appears to vary by clinical specialties, with pediatric notes containing the greatest amount of redundant information (75%).

## Abbreviations

DO: Document Ontology
KOD: Kind of Document
LCS: Least Common Subsume
LVG: Lexical Variant Generation
RTF: Rich Text Format
SLM: Statistical language modeling
SMD: Subject Matter Domains
TF-IDF: Term Frequency - Inverse Document Frequency
TOS: Type of Service
UMLS: Unified Medical Language System

## References

[1] Hirschtick RE (2006). A piece of my mind. Copy-and-paste. JAMA.

[2] Markel A (2010). Copy and paste of electronic health records: a modern medical illness. Am J Med.

[3] Bowman S (2013). Impact of Electronic Health Record Systems on Information Integrity: Quality and Safety Implications. Perspect Health Inf Manag.

[4] Farri O, Pieckiewicz DS, Rahman AS, Adam TJ, Pakhomov SV, Melton GB (2012). A qualitative analysis of EHR clinical document synthesis by clinicians. AMIA Annu Symp Proc.

[5] Farri O (2012). Impact of a prototype visualization tool for new information in EHR clinical documents. Appl Clini Inform.

[6] Hersh W (2007). Copy and Paste. AHRQ WebM&M.

[7] Hripcsak G, Vawdrey DK, Fred MR, Bostwick SB (2011). Use of electronic clinical documentation: time spent and team interactions. J Am Med Inform Assoc.

[8] Cohen R, Elhadad M, Elhadad N (2013). Redundancy in electronic health record corpora: analysis, impact on text mining performance and mitigation strategies. BMC Bioinforma.

[9] Pivovarov R, Elhadad N (2015). Automated methods for the summarization of electronic health records. J Am Med Inform Assoc.

[10] Zhang R, Pakhomov S, Melton GB (2014). Longitudinal analysis of new information types in clinical notes. AMIA Jt Summits Transl Sci Proc.

[11] Weir CR, Hurdle JF, Felgar MA, Hoffman JM, Roth B, Nebeker JR (2003). Direct text entry in electronic progress notes. An evaluation of input errors. Methods Inf Med.

[12] Hammond KW, Helbig ST, Benson CC, Brathwaite-Sketoe BM (2003). Are electronic medical records trustworthy? Observations on copying, pasting and duplication. AMIA Annu Symp Proc..

[13] Wrenn JO, Stein DM, Bakken S, Stetson PD (2010). Quantifying clinical narrative redundancy in an electronic health record. J Am Med Inform Assoc.

[14] Zhang R, Pakhomov S, McInnes BT, Melton GB (2011). Evaluating Measures of Redundancy in Clinical Texts. AMIA Annu Symp Proc.

[15] Zhang R, Pakhomov S, Melton GB (2012). Automated Identification of Relevant New Information in Clinical Narrative. IHI’12 ACM Interna Health Inform Sym Proc.

[16] Zhang R, Pakhomov S, Lee JT, Melton GB (2013). Navigating longitudinal clinical notes with an automated method for detecting new information. Stud Health Technol Inform.

[17] McCray AT, Burgun A, Bodenreider O (2001). Aggregating UMLS semantic types for reducing conceptual complexity. Stud Health Technol Inform.

[18] Manning CD, SchÜtze H (2003). Foundations of Statistical Natural Language Processing.

[19] Jurafsky D, Martin JH (2009). Speech and Language Processing.

[20] Rada R, Mili H, Bicknell E, Blettner M (1989). Development and Application of a Metric on Semantic Nets. IEEE Trans Syst Man Cybern.

[21] Caviedes JE, Cimino JJ (2004). Towards the development of a conceptual distance metric for the UMLS. J Biomed Inform.

[22] Lord PW, Stevens RD, Brass A, Goble CA (2003). Investigating semantic similarity measures across the Gene Ontology: the relationship between sequence and annotation. Bioinformatics.

[23] Pedersen T, Pakhomov SV, Patwardhan S, Chute CG (2007). Measures of semantic similarity and relatedness in the biomedical domain. J Biomed Inform.

[24] McInnes B, Pedersen T, Pakhomov S (2009). UMLS-Interface and UMLS-Similarity : open source software for measuring paths and semantic similarity. AMIA Annu Symp Proc.

[25] Resnik P (1995). Using Information Content to Evaluate Semantic Similarity in a Taxonomy. International Joint Conference for Artificial Intelligence.

[26] Jiang J, Conrath D (1997). Semantic similarity based on corpus statistics and lexical taxonomy. Proceedings on International Conference on Research in CL.

[27] Lin D (1998). An information-theoretic definition of similarity. Proceedings of the International Conference on ML.

[28] McInnes B, Pedersen T, Liu Y, Melton GB, Pakhomov S (2011). Knowledge-based Method for Determining the Meaning of Ambiguous Biomedical Terms Using Information Content Measures of Similarity. AMIA Annu Symp Proc..

[29] Dolin RH (2006). HL7 Clinical Document Architecture, Release 2. J Am Med Inform Assoc.

[30] Shapiro JS, Bakken S, Hyun S, Melton GB, Schlegel C and Johnson SB. Document ontology: supporting narrative documents in electronic health records. AMIA Annu Symp Proc. 2005;684-8. PMC1560738 16779127

[31] Wang Y, Pakhomov S, Dale JL, Chen ES, Melton GB (2014). Application of HL7/LOINC Document Ontology to a University-Affiliated Integrated Health System Research Clinical Data Repository. AMIA Jt Summits Transl Sci Proc.

[32] Zhang R, Pakhomov S, Lee J, Melton GB (2014). Using Language Models to Identify Relevant New Information in Inpatient Clinical Notes. Proc AMIA Symp.

[33] *Stopword List*. Available: http://www.textfixer.com/resources/common-english-words.txt. Accessed May 2017.

[34] Aronson AR, Lang FM (2010). An overview of MetaMap: historical perspective and recent advances. J Am Med Inform Assoc.

[35] Zhang R, Pakhomov SV, Lee J, Melton GB (2014). Using Language Models to Identify Relevant New Information in Inpatient Clinical Notes. AMIA Annu Symp Proc.

